# The efficacy of additional surgical resection after endoscopic resection in pT1b esophageal squamous cell carcinoma: A multi-institutional retrospective study in China

**DOI:** 10.1007/s00464-022-09459-5

**Published:** 2022-08-25

**Authors:** Xuemin Xue, Qi Sun, Dongxian Jiang, Xinran Wang, Yong Liu, Changyuan Guo, Linxiu Liu, Na Cheng, Guiqi Wang, Yueping Liu, Yingyong Hou, Xiangshan Fan, Liyan Xue

**Affiliations:** 1 Department of Pathology, National Cancer Center/National Clinical Research Center for Cancer/Cancer Hospital, Chinese Academy of Medical Sciences and Peking Union Medical College, Beijing, 100021 China; 2 Department of Pathology, The Affiliated Drum Tower Hospital of Nanjing University Medical School, Nanjing, 210008 Jiangsu Province China; 3 Department of Pathology, Zhongshan Hospital, Fudan University, Shanghai, 200032 China; 4 Department of Pathology, The Fourth Hospital of Hebei Medical University, Shijiazhuang, 050011 Hebei Province China; 5 Department of Endoscopy, National Cancer Center/National Clinical Research Center for Cancer/Cancer Hospital, Chinese Academy of Medical Sciences and Peking Union Medical College, Beijing, 100021 China

**Keywords:** Esophageal squamous cell carcinoma, Endoscopic resection, Surgical resection, Survival

## Abstract

**Background:**

pT1b esophageal squamous cell carcinoma (ESCC) patients treated by endoscopic resection (ER) required additional treatment with surgical resection (SR) or chemoradiotherapy (CRT) according to 2020 Japan Gastroenterological Endoscopy Society (JGES) guideline. Given the evidences for this recommendation were largely based on small-size studies, our study collected 166 cases of ER-treated pT1b patients in order to investigate the efficacy of additional SR as compared to ER-alone treatment.

**Methods:**

A multi-institutional retrospective study in China was conducted. The pT1b ESCC treated by ER + SR (*n* = 42) and ER-alone (*n* = 124) from 2007 to 2018 were recruited. Meanwhile, patients with positive lymphovascular invasion (LVI(+)) and/or with positive vertical margin (VM(+)) were put into high-risk group, and those with both VM(−) and LVI(−) were selected into low-risk group. The clinicopathological parameters, lymph node metastasis (LNM), and survival between ER + SR and ER-alone groups were analyzed.

**Results:**

In high-risk group, concurrent LNM revealed in surgically resected specimens accounted for 52.6% cases in ER + SR group. After surgical removal, the incidence of post-resection LNM dropped down to 5.6%. However, in low-risk group, patients with ER + SR treatment did not exhibit any concurrent LNM in surgically resected specimens, and the incidence of their overall LNM was similar to that in ER-alone group (0% vs. 2.8%, *p* = 1.000). More importantly, these cases demonstrated significantly shorter overall survival (OS) than that in ER-alone group (81.8% and 100.0%, respectively, at 3 years; log-Rank: *P* = 0.010).

**Conclusions:**

For ER-treated pT1b patients in high-risk group, additional SR is strongly recommended. However, for those in low-risk group, additional SR does not generate much benefit for clearance of LNM, but brings harm to shorten their OS. Therefore, additional SR is not recommended for ER-treated pT1b patient in low-risk group.

Endoscopic resection (ER) has become gold standard for the treatment of superficial esophageal squamous cell carcinoma (ESCC) due to safe, short recovery time, and the preservation of esophagus, as compared to esophageal radical surgical resection (SR). According to 2020 Japan Gastroenterological Endoscopy Society (JGES) guideline for esophageal cancer, additional treatments with surgical resection or chemoradiotherapy (CRT) are strongly recommended for pT1b ESCC based on post-ER pathological report [1]. However, given the evidences were largely originated from small-size retrospective studies and some of them were based on non-endoscopic studies, this recommendation has not been sufficiently investigated [2–7].

Data from radical SR demonstrated that the incidences of lymph node metastasis (LNM) in ESCC with sm1, sm2, and sm3 invasion (superficial, middle, and deep thirds of the submucosa, respectively) were16.92–24.0%, 13.73–20.5%, and 34.3–43.8%, respectively [4, 7, 8]. Due to the stringent selection criteria, such as no obvious LNM and no clear submucosal infiltration revealed by pre-ER imaging and endoscopic examinations, ER-treated pT1b ESCC patients demonstrate relatively less aggressive clinical course than those SR-treated pT1b counterparts [1]. Thus, we were interested in seeing if there was a way to optimize the benefit-to-harm balance of additional treatment, especially additional SR. Herein, our study collected 166 cases of ER-treated pT1b patients in order to investigate the efficacy of additional SR in low- and high-risk groups of ESCC.

## Materials and methods

### Patients

**Fig. 1:**
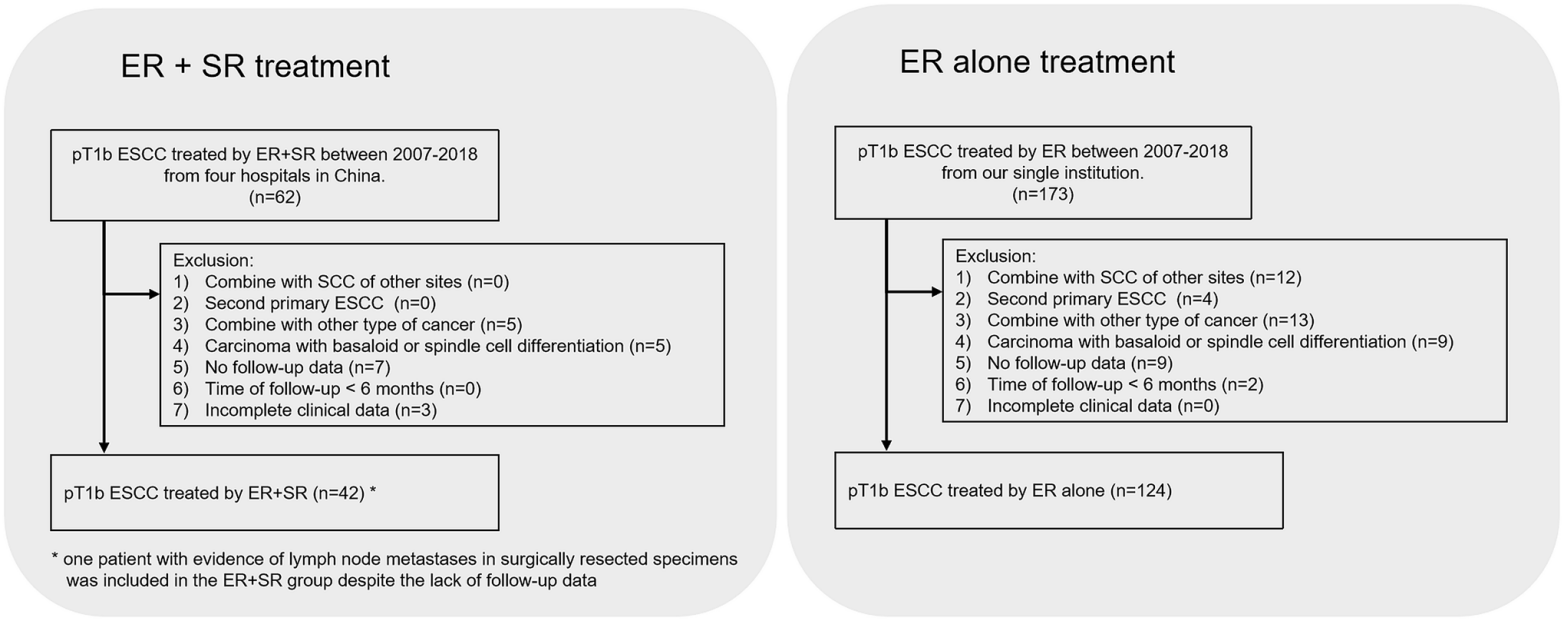
Flowchart of the study design. *ER* endoscopic resection, *SR* surgical resection, *SCC* squamous cell carcinoma, *ESCC* esophageal squamous cell carcinoma. One patient with evidence of lymph node metastases in surgically resected specimens was included in the ER + SR group despite the lack of follow-up data

This was a multi-institutional retrospective research carried out at four institutions in China. We retrospectively recruited 62 consecutive pT1b patients treated by ER plus SR (esophagectomy + two-field lymphadenectomy) from 2007 to 2018. They did not undergo any preoperative/postoperative radio chemotherapy. We excluded cases with (1) Combination with squamous cell carcinoma of other sites, (2) Second primary ESCC, (3) Combination with other type of cancer, (4) Carcinoma with basaloid or spindle cell differentiation, (5) No follow-up data, (6) Time of follow-up < 6 months, and (7) Incomplete clinical data (e.g., no SMI depth value) (Fig. 1). It should be noted that one patient with evidence of lymph node metastases in surgically resected specimens was included in the ER + SR group despite the lack of follow-up data. Ultimately, 42 cases with ER + SR treatment were analyzed from four hospitals, including National Cancer Center/National Clinical Research Center for Cancer/Cancer Hospital(*n* = 20), Nanjing Drum Tower Hospital (*n* = 8), Shanghai Zhongshan Hospital (*n* = 8), and the Fourth Hospital of Hebei Medical University (*n* = 6) (Fig. 1).

Meanwhile, we also retrospectively collected 173 consecutive pT1b ESCC patients with ER-alone treatment between 2007 and 2018 from single institution (National Cancer Center/National Clinical Research Center for Cancer/Cancer Hospital). Based on the same exclusion criterion mentioned previously in ER + SR group, 124 cases were finally analyzed in ER-alone group (Fig. 1). These pT1b patients refused surgery despite knowing the risk of LNM. They all did not undergo any preoperative/postoperative radio chemotherapy.

### Data collection

The clinicopathological data in our study were collected from medical record at each institution. The information regarding tumor location, histological type, tumor differentiation, lymphovascular invasion (LVI), vertical margin (VM), and submucosal invasion (SMI) were generated from endoscopically resected specimens. The treatment and follow-up data were acquired by means of medical record consultation and telephone conversation. Poor differentiation (PD) of tumor was defined according to the 5th edition of the WHO Classification of Tumors of Digestive System Tumors [9]. It is important to note that carcinoma with basaloid or spindle cell differentiation was excluded in our study.

In ER + SR group, concurrent LNM was defined when LNM was found in surgically resected specimens. Post-resection LNM and distant organ metastasis (DOM) were defined when metastasis was detected after SR. The overall LNM is composed of concurrent and post-resection LNM in ER + SR group.

Similarly, in ER-alone group, post-resection LNM and DOM were defined when metastasis was detected after ER. Because of no additional SR in this group, none of the concurrent LNM data were generated. Thus, the overall LNM is composed of only post-resection LNM in ER-alone group.

### Statistical analysis

The times of overall survival (OS) and progression-free survival (PFS) were defined from the date of ER to the date of death/event or the last follow-up. The day of last follow-up was January 9^st^ 2021. The survival curves were plotted by Kaplan–Meier procedure with log-rank test.

For comparison of pathological characteristics between two groups, three different statistical tests were used according to the type of variables. Mann–Whitney test was used for continuous variables, such as age. χ^2^ test was used for categorical variable, such as sex and VM. Fisher’s exact test was typically used as an alternative to the χ^2^ test when one or more of the cell counts in a 2 × 2 table is less than 5.

All statistical analyses were two sided and *p* < 0.05 was defined as significance. All the above statistical analyses were run in R 3.6.0 statistical software.

## Results

### *The clinicopathological characteristics of pT1b ESCC between ER* + *SR and ER-alone groups*

The clinicopathological characteristics of pT1b ESCC between ER + SR and ER-alone groups are summarized in Table 1. The median time of follow-up was 38.1 months (range 6.0–136.9 months) for all patients. It should be noted that one case in ER + SR group demonstrating concurrent LNM in surgically resected specimens was also included in the analysis despite the absence of follow-up data. Thus, the incidence of overall LNM for total patients was 9.0% (15/166), and the incidences of post-resection LNM and DOM for all cases were 3.6% (6/165) and 7.3% (12/165), respectively (Table 1).

**Table 1:** The clinicopathological characteristics of pT1b ESCC between ER + SR and ER-alone groups

		ER + SR	ER-alone	*p* value (*χ*^2^ test)
		(*n* = 42)	(*n* = 124)	
Age				
	Median [IQR]	60.5 [55.00, 64.00]	62 [57.00, 67.00]	0.101^#^
Sex				
	Female	5 (11.9%)	31 (25.0%)	0.118
	Male	37 (88.1%)	93 (75.0%)	
SMI ≥ 200 μm				
	Negative	5 (11.9%)	33 (26.6%)	0.080
	Positive	37 (88.1%)	91 (73.4%)	
VM				
	Negative	32 (76.2%)	121 (97.6%)	< 0.001^^^
	Positive	10 (23.8%)	3 (2.4%)	
LVI				
	Negative	29 (69.0%)	108 (87.1%)	0.015
	Positive	13 (31.0%)	16 (12.9%)	
PD				
	Negative	30 (71.4%)	72 (58.1%)	0.176
	Positive	12 (28.6%)	52 (41.9%)	
Location				
	Upper	7 (16.7%)	17 (13.7%)	0.319
	Middle	9 (21.4%)	42 (33.9%)	
	Lower	26 (61.9%)	65 (52.4%)	
Concurrent LNM				
	Negative	32 (76.2%)	NA	NA
	Positive	10 (23.8%)	NA	
Post-resection LNM				
	Negative	40 (97.6%)	119 (96.0%)	1.000^^^
	Positive	1 (2.4%)^a^	5 (4.0%)	
Overall LNM				
	Negative	32 (76.2%)	119 (96.0%)	< 0.001
	Positive	10 (23.8%)	5 (4.0%)	
Post-resection DOM				
	Negative	36 (87.8%)	117 (94.4%)	0.292
	Positive	5 (12.2%)	7 (5.6%)	

*IQR* interquartile range; *PD* poor differentiation; *LVI* lymphovascular invasion; *VM* vertical margin; *SMI* Submucosal invasion; *LNM* lymph node metastasis; *DOM* distant organ metastasis; *NA* not available

^#^Mann–Whitney test

^^^Fisher’s exact test

^a^One case with ER + SR treatment revealed both concurrent and post-resection LNM

Furthermore, one case with ER + SR treatment revealed both concurrent and post-resection LNM. Thus, the incidence of post-resection LNM was 2.4% (1/41), and the incidences of concurrent and overall LNM in ER + SR group were both 23.8% (10/42) (Table 1).

**Fig. 2:**
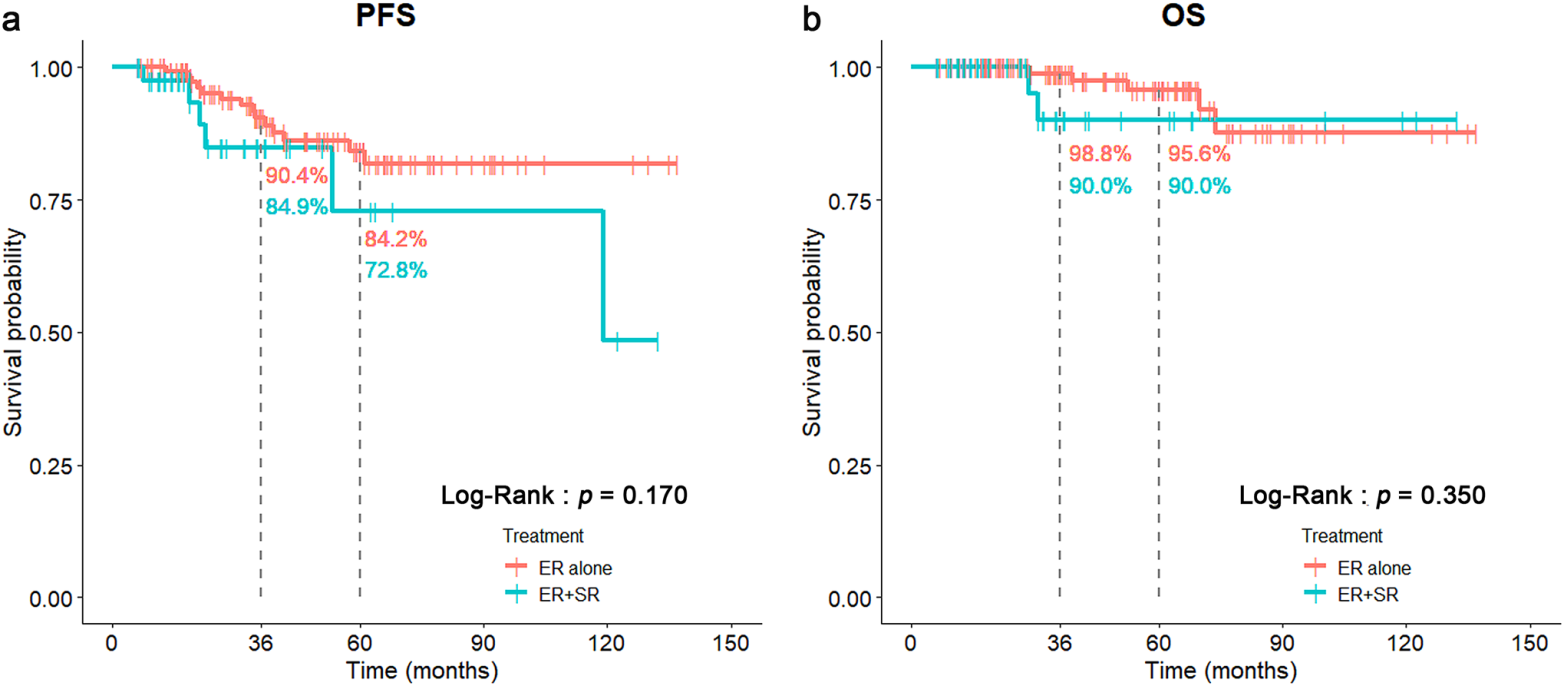
Comparisons of PFS (**a**) and OS (**b**) between ER-alone and ER + SR treatment

Moreover, the incidences of LVI( +)(31.1%) and VM( +)(23.8%) in ER + SR group were significantly higher than those in ER-alone group (Table 1). The overall LNM in ER + SR group was also significantly higher than those in ER-alone group (23.8% vs. 4.0%, *p* < 0.001) (Table 1). However, the post-resection LNM did not demonstrate any significance (Table 1). The OS and PFS between these two groups also did not reveal any significant difference (*p* = 0.350 and 0.170, respectively) (Fig. 2).

### *The comparisons of clinicopathological characteristics between ER* + *SR and ER-alone groups with LVI(* +*) and/or VM(* +*)*

Based on the status of LVI and VM, the patients were divided into low- and high-risk groups. Patients with LVI( +) and/or VM( +) were selected into the high-risk group, and patients with both LVI(−) and VM(−) were put into low-risk group.

**Fig. 3:**
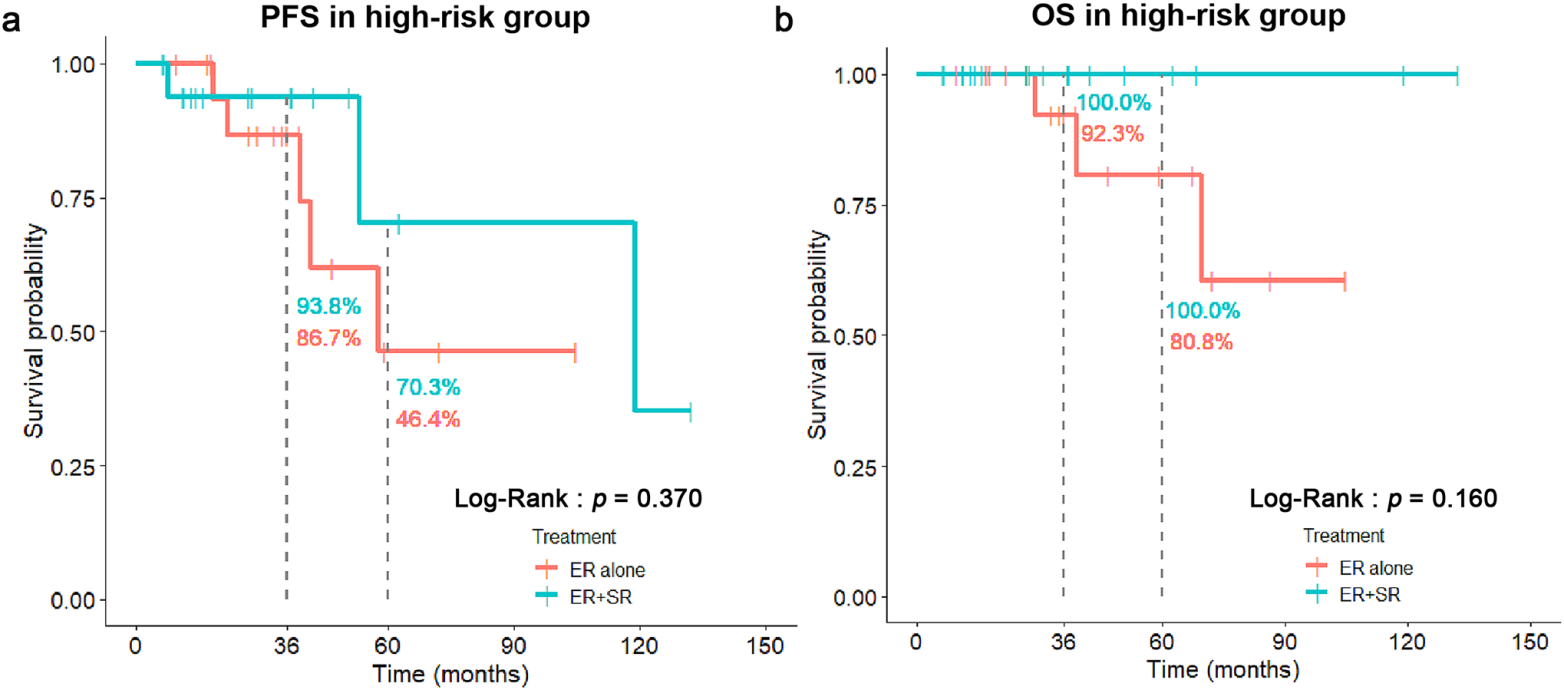
Comparisons of PFS (**a**) and OS (**b**) between ER-alone and ER + SR treatment in high-risk group with LVI( +) and/or VM( +)

For patients with LVI( +) and/or VM( +), concurrent LNM in surgically resected specimens was detected in 52.6% cases of patients with ER + SR treatment. After radical surgical removal, the incidence of post-resection LNM dropped down to 5.6% (Table 2). Thus, no significant difference in post-resection LNM and DOM was found between ER + SR and ER-alone groups (Table 2). The OS and PFS between these two groups also did not show any significant difference (*p* = 0.160 and 0.370, respectively) (Fig. 3).

**Table 2:** The comparisons of clinicopathological characteristics between ER + SR and ER-alone treatments in high-risk group with LVI(+) and/or VM(+)

		ER + SR	ER-alone	*p* value (*χ*^2^ test)
		(*n* = 19)	(*n* = 18)	
Age				
	Median [IQR]	62.00 [55.00, 63.50]	61.00 [54.00, 69.75]	0.522^#^
Sex				
	Female	1 (5.3%)	2 (11.1%)	0.604^^^
	Male	18 (94.7%)	16 (88.9%)	
SMI ≥ 200 μm				
	Negative	0 (0.0%)	4 (22.2%)	0.046^^^
	Positive	19 (100.0%)	14 (77.8%)	
VM				
	Negative	9 (47.4%)	15 (83.3%)	0.038^^^
	Positive	10 (52.6%)	3 (16.7%)	
LVI				
	Negative	6 (31.6%)	2 (11.1%)	0.232^^^
	Positive	13 (68.4%)	16 (88.9%)	
PD				
	Negative	9 (47.4%)	9 (50.0%)	1.000
	Positive	10 (52.6%)	9 (50.0%)	
Location				
	Upper	1 (5.3%)	2 (11.1%)	0.048
	Middle	1 (5.3%)	6 (33.3%)	
	Lower	17 (89.5%)	10 (55.6%)	
Concurrent LNM				
	Negative	9 (47.4%)	NA	NA
	Positive	10 (52.6%)	NA	
Post-resection LNM				
	Negative	17 (94.4%)	16 (88.9%)	1.000^^^
	Positive	1 (5.6%)^a^	2 (11.1%)	
Overall LNM				
	Negative	9 (47.4%)	16 (88.9%)	0.013^^^
	Positive	10 (52.6%)	2 (11.1%)	
Post-resection DOM				
	Negative	16 (88.9%)	17 (94.4%)	1.000^^^
	Positive	2 (11.1%)	1 (5.6%)	

*IQR* interquartile range; *PD* poor differentiation; *LVI* lymphovascular invasion; *VM* vertical margin; *SMI* Submucosal invasion; *LNM* lymph node metastasis; *DOM* distant organ metastasis; *NA* not available

^#^Mann–Whitney test

^^^Fisher’s exact test

^a^One case with ER + SR treatment revealed both concurrent and post-resection LNM

### *The comparisons of clinicopathological characteristics between ER* + *SR and ER-alone groups with both LVI(−) and VM(−)*

**Fig. 4:**
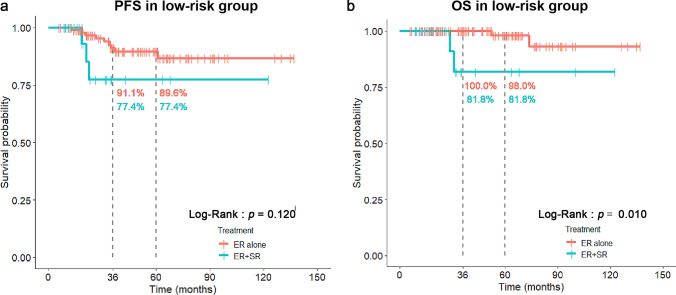
Comparisons of PFS (**a**) and OS (**b**) between ER-alone and ER + SR treatment in low-risk group with both LVI(−) and VM(−)

In low-risk group with both LVI(−) and VM(−), patients with ER + SR treatment demonstrated significantly worse OS than those with ER-alone treatment (81.8% and 100.0% at 3 years, respectively; Log-Rank: *p* = 0.010). However, no significant difference in PFS was found between these two groups (77.4% and 91.1% at 3 years, respectively; Log-Rank: *p* = 0.120) (Fig. 4). The incidence of overall LNM in ER + SR group was similar to that in ER-alone group (0% vs. 2.8%, *p* = 1.000) (Table 3). More importantly, none of concurrent LNM in surgically resected specimens was found in patients with ER + SR treatment (Table 3).

**Table 3:** The comparisons of clinicopathological characteristics between ER + SR and ER-alone treatments in low-risk group with both LVI(−) and VM(−)

		ER + SR	ER-alone	*p_value* (χ^2^ test)
		(*n* = 23)	(*n* = 106)	
Age				
	Median [IQR]	60.00 [55.00, 65.00]	62.00 [57.00, 67.00]	0.201^#^
Sex				
	Female	4 (17.4%)	29 (27.4%)	0.432^^^
	Male	19 (82.6%)	77 (72.6%)	
SMI ≥ 200 μm				
	Negative	5 (21.7%)	29 (27.4%)	0.769
	Positive	18 (78.3%)	77 (72.6%)	
PD				
	Negative	21 (91.3%)	63 (59.4%)	0.003^^^
	Positive	2 (8.7%)	43 (40.6%)	
Location				
	Upper	6 (26.1%)	15 (14.2%)	0.320
	Middle	8 (34.8%)	36 (34.0%)	
	Lower	9 (39.1%)	55 (51.9%)	
Concurrent LNM				
	Negative	23 (0%)	NA	NA
	Positive	0 (0%)	NA	
Post-resection LNM				
	Negative	23 (100.0%)	103 (97.2%)	1.000^^^
	Positive	0 (0.0%)	3 (2.8%)	
Overall LNM				
	Negative	23 (100.0%)	103 (97.2%)	1.000^^^
	Positive	0 (0.0%)	3 (2.8%)	
Post-resection DOM				
	Negative	20 (87.0%)	100 (94.3%)	0.200^^^
	Positive	3 (13.0%)	6 (5.7%)	

*IQR* interquartile range; *PD* poor differentiation; *LVI* lymphovascular invasion; *VM* vertical margin; *SMI* Submucosal invasion; *LNM* lymph node metastasis; *DOM* distant organ metastasis; *NA* not available

^#^Mann–Whitney test

^^^Fisher’s exact test

### *The comparisons of clinicopathological characteristics between ER* + *SR and ER-alone treatments in extremely low-risk group with VM(−), LVI(−), and PD(−)*

**Fig. 5:**
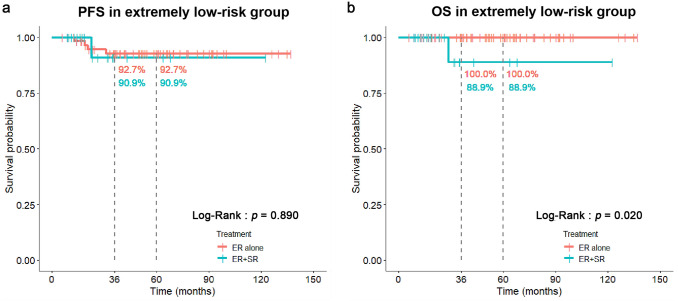
Comparisons of PFS (**a**) and OS (**b**) between ER-alone and ER + SR treatment in extremely low-risk group with LVI(−), VM(−), and PD(−)

In order to eliminate potential impacts by PD, we identified an extremely low-risk group with VM(−), LVI(−), and PD(−). The results showed that patients with ER + SR treatment still had significantly worse OS than those with ER-alone treatment (88.9% and 100.0% at 3 years, respectively; Log-Rank: *P* = 0.020). However, no significant difference in PFS was found between these two treatments (90.9% and 92.7% at 3 years, respectively; Log-Rank: *p* = 0.890) (Fig. 5). More importantly, none of concurrent LNM in surgically resected specimens was found in patients with ER + SR treatment (Table 4).

**Table 4:** The comparisons of clinicopathological characteristics between ER + SR and ER-alone treatments in extremely low-risk group with VM(−), LVI(−), and PD(−)

		ER + SR	ER-alone	*p_value* (χ^2^ test)
		(*n* = 21)	(*n* = 63)	
Age				
	Median [IQR]	60.00 [55.00, 65.00]	63.00 [57.50, 67.00]	0.382^#^
Sex				
	Female	4 (19.0%)	15 (23.8%)	0.770^^^
	Male	17 (81.0%)	48 (76.2%)	
SMI ≥ 200 μm				
	Negative	5 (23.8%)	14 (22.2%)	1.000
	Positive	16 (76.2%)	49 (77.8%)	
Location				
	Upper	6 (28.6%)	10 (15.9%)	0.437
	Middle	7 (33.3%)	24 (38.1%)	
	Lower	8 (38.1%)	29 (46.0%)	
Concurrent LNM				
	Negative	21 (100%)	NA	NA
	Positive	0 (0%)	NA	
Post-resection LNM				
	Negative	21 (100.0%)	61 (96.8%)	1.000^^^
	Positive	0 (0.0%)	2 (3.2%)	
Overall LNM				
	Negative	21 (100.0%)	61 (96.8%)	1.000^^^
	Positive	0 (0.0%)	2 (3.2%)	
Post-resection DOM				
	Negative	20 (95.2%)	61 (96.8%)	1.000^^^
	Positive	1 (4.8%)	2 (3.2%)	

*IQR* interquartile range; *PD* poor differentiation; *LVI* lymphovascular invasion; *VM* vertical margin; *SMI* Submucosal invasion; *LNM* lymph node metastasis; *DOM* distant organ metastasis; *NA* not available

^#^Mann–Whitney test

^^^Fisher’s exact test

## Discussion

In this multicenter retrospective study, the incidences of overall LNM and post-resection DOM for total pT1b patients were only 9.0% (15/166) and 7.2% (12/166), respectively. Because of the strict selection by pre-ER imaging and endoscopic examination, LNM was exhibited to be less frequent in ER-treated pT1b ESCC shown in our study than in SR-treated counterparts reported in the literatures [4, 7, 8]. Thus, ER-treated pT1b ESCC indicated a less aggressive subset of pT1b ESCC. However, according to 2020 JGES guideline, this subgroup required additional treatment with SR or CRT [1]. Given the evidences for this recommendation were largely based on several small-size retrospective studies, our study enrolled 42 patients with ER + SR treatment and 124 cases with ER-alone treatment to evaluate the effectiveness of additional SR, aiming to explore the benefit-to-harm balance of additional SR for ER-treated pT1b ESCC.

Except for those patients who refused surgery despite understanding the risk of LNM, ER-treated pT1b patients with high-risk pathological factors tend to receive additional SR according to the currently curative criteria [1, 3, 6, 10]. Thus, as what we expected, VM( +) and LVI( +) in ER + SR group were significantly higher than those in ER-alone group (Table 1). Consequently, the incidence of overall LNM in ER + SR group was also significantly greater than that in ER-alone group (23.8% vs. 4.0%, *p* < 0.001) (Table 1). But the good news was that, after surgical removal of concurrent LNM which accounted for 23.8% cases in ER + SR group, the incidences of post-resection LNM dropped down to 2.4% (Table 1). Even more obviously, for those patients in high-risk group with LVI( +) and/or VM( +), concurrent LNM accounted for 52.6% cases in ER + SR group. After surgical clearance, the incidences of post-resection LNM and DOM were reduced to 5.6% and 11.1%, respectively (Table 2). All these results indicated the necessity of additional SR for pT1b ER-treated patients in high-risk group, overcoming their adverse effects of metastasis. Thus, the recommendation of additional SR for patients with LVI( +) and/or VM( +) is not in question [1, 10].

However, we were more curious about the effectiveness of additional SR in low-risk group who had both LVI(−) and VM(−). For these pT1b patients in our study, the incidence of overall LNM in ER + SR group was similar to that in ER-alone group (0% vs. 2.8%, *p* = 1.000) (Table 3). More importantly, none of concurrent LNM in surgically resected specimens was found in ER + SR group (Table 3), which indicated that additional SR in low-risk group does not generate much benefit for clearance of LNM. Instead, it may bring procedure-related complications or death to patients[11], since significantly shorter OS was demonstrated in ER + SR group as compared to that in ER-alone group (81.8% and 100.0% at 3 years, respectively; Log-Rank: *P* = 0.010) (Fig. 4). The similar results were found in extremely low-risk group who had VM(−), LVI(−), and PD(−) (Fig. 5).Therefore, based on our findings, additional SR is not the best selection for those ER-treated pT1b patients in low-risk group. Adjuvant chemoradiotherapy may be a reasonable option [11–14].

To the best of our knowledge, this is the largest study to evaluate the efficacy of additional SR for ER-treated patients. But some statistical bias may be involved, such as retrospective study and a relatively short follow-up period, which requires further investigation.

In conclusion, for ER-treated pT1b patients in high-risk group, additional SR is strongly recommended. However, for those in low-risk group, additional SR does not generate much benefit for the removal of LNM, but brings harm of procedure-related complications or death to shorten their OS. Therefore, additional SR is not recommended for patient with both VM(−) and LVI(−).
